# Mechanical characterization of vertically aligned carbon nanotube forest microelectrodes for neural interfacing

**DOI:** 10.3389/fnins.2026.1864874

**Published:** 2026-07-16

**Authors:** Spencer M. Roberts, Joseph S. Carter, Brian D. Jensen, Richard R. Vanfleet, Robert C. Davis

**Affiliations:** 1 Department of Physics and Astronomy, Brigham Young University, Provo, UT, United States; 2 Department of Mechanical Engineering, Brigham Young University, Provo, UT, United States

**Keywords:** carbon nanotube forest, finite element analysis, mechanical characterization, microelectrode array, modulus, neural implant

## Abstract

**Introduction:**

This study aims to explore the mechanical properties of porous microelectrodes formed from vertically aligned carbon nanotube (CNT) forests. Specifically, we investigate the range of effective CNT-based microelectrode (ME) moduli that can be fabricated and identify moduli within that range that significantly reduce strain on brain tissue during micromotion.

**Materials and methods:**

To address these questions, we developed a micromechanical measurement method, known as the dual deflection (DD) test, which is compatible with microelectrode array (MEA) form factors and can measure a wide range of moduli with a 30% uncertainty. Using the DD test with small deflections, we measured the effective Young’s modulus of freestanding CNT microelectrodes (MEs) fabricated with different carbon infiltration times (0, 15, and 30 s) at 900°C. We also developed a static 10 μm deflection finite element analysis (FEA) model to compare the brain tissue strain induced by probes with the maximum (1.7 GPa), median (72 MPa), and minimum (3.9 MPa) measured CNT moduli, along with the modulus of silicon (165 GPa) for comparison.

**Results:**

The DD test results showed mean effective moduli of 19.6 ± 14.5 MPa, 67.7 ± 22.7 MPa, and 168 ± 62.3 MPa for arrays fabricated with 0, 15, and 30 s infiltrations, respectively. The FEA model revealed that probes with the maximum CNT modulus induced similar strain to the silicon probes at the tip, while probes with the minimum and median CNT moduli showed minimal strain at the tip.

**Discussion:**

These findings suggest that CNT microelectrodes with moduli in the tens of MPa range, achievable through 15 s of carbon infiltration, can significantly reduce brain tissue strain. Additionally, we consistently observed that microelectrodes with 15 s of infiltration were apparently undamaged after deflection, making them mechanically promising candidates for neural probe arrays.

## 1 Introduction

Brain computer interfaces (BCIs) hold the potential to revolutionize treatment for individuals afflicted with neurological disorders, amputations, and spinal cord injuries. Successful clinical implementation of BCIs requires intracortical microelectrode arrays (MEAs) that can provide high spatial and temporal resolutions of neural signals, along with a high signal-to-noise ratio (SNR). However, clinical intracortical MEA devices must satisfy the requirements with high-performance specifications for an extended period—ideally a decade—which has yet to be achieved with current technologies (Krüger et al., 2010; Joshi-Imre et al., 2019; George et al., 2020; Hong et al., 2021; Sponheim et al., 2021; Yang et al., 2021). Although no intracortical implant is presently suitable for clinical use, the Utah Electrode Array (UEA) has been the gold standard for intracortical MEAs since its development in the 1990s (Normann et al., 1996; Rousche and Normann, 1998; Branner et al., 2004; Leber et al., 2019). To date, the UEA is the only intercortical MEA with FDA 510(k) clearance that has been utilized in long-term non-human and human implantation studies (Leber et al., 2019; U.S. Food and Drug Administration, 2022). A meta-analysis conducted by Sponheim et al. (2021) on all available *in vivo* studies utilizing the UEA revealed that out of 55 arrays, 16 successfully maintained an electrode yield of 40% or higher for over 800 days, with three arrays lasting more than 5 years *in vivo* (Sponheim et al., 2021). In this analysis, individual electrodes were considered “good” and contributed to the yield if they exceeded an SNR of 1.5. Notably, two of the three long-lasting arrays were implanted in human subjects. However, despite a few instances of extended long-term recordings, 68% of the 51 implantations in non-human primates had to be explanted due to signal loss. Sponheim et al. observed that the arrays typically experienced a ∼2% decrease in yield every 30 days, resulting in only 50% of viable arrays remaining after 1 year of implantation. Although the lifetime yields of the UEA are among the highest reported, they still fall short of clinical specifications, indicating that there is significant potential for lifetime improvement with UEA-style implants.

**FIGURE 1:**
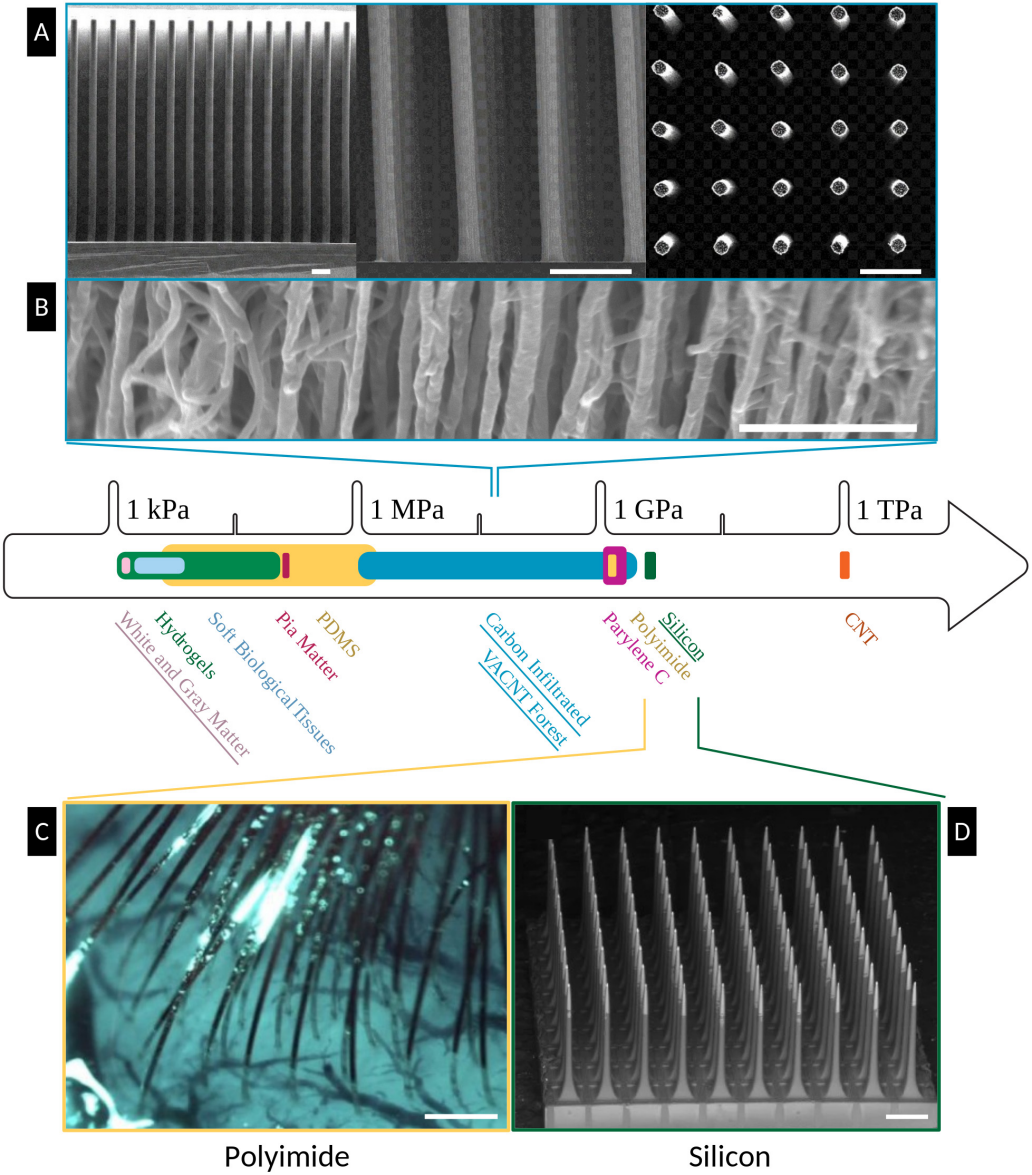
The large range of Young’s moduli for materials relevant to neural implants with example devices. **(A)** An electrode array fabricated from a carbon infiltrated vertically aligned carbon nanotube (CI-VACNT) forest. All scale bars are 400 μm. Adapted with permission from Chen et al. (2018). Copyright © 2018 American Chemical Society. **(B)** A close-up SEM image of the carbon infiltrated VACNT forest. Scale bar is 500 nm. **(C)** Polyimide neural array produced by Neuralink after implantation in cortex. Scale bar is 500 μm. Adapted with permission from Musk and Neuralink (2019). Copyright © 2019 Musk and Neuralink. **(D)** Silicon Utah electrode array (UEA). Scale bar is 400 μm. Reproduced with permission from Bhandari et al. (2010). Copyright © 2010 Springer Nature.

A hypothesized factor contributing to the long-term decline in yield of MEA electrodes is inadequate biocompatibility. An implant is considered to have poor biocompatibility when the chronic foreign body response (FBR) induced by the implant significantly hinders its functionality (Williams, 2008). The chronic FBR is exacerbated by the mechanical mismatch between the probe and the surrounding tissue during brain micromotion. This micromotion can be caused by factors such as respiration, pulsation, and rotational acceleration of the head, which can displace a penetrating electrode by tens of microns relative to the nearby tissue (Lee et al., 2005; Gilletti and Muthuswamy, 2006). During this motion, the large mechanical mismatch between the stiff silicon electrode (∼165 GPa) and the soft cortical brain tissue (∼1–10 kPa) (Figure 1) can induce significant strain on the brain, which activates the chronic immune response and may even lead to neural tissue failure if > 0.1 in tension and 0.3 in compression (Lee et al., 2005; Biran et al., 2007; Bilston, 2011; Moshayedi et al., 2014; Mian et al., 2021; Wu et al., 2021; Zhang D. et al., 2021; Sharafkhani et al., 2022). Lowering the strain can diminish the foreign body response, improve the signal-to-noise ratio, and extend the functional lifespan of the probe (Lecomte et al., 2018; Stiller et al., 2018). Researchers have explored the use of soft, biocompatible encapsulations and coatings on MEAs to minimize the mismatch at the tissue-electrode interface, but to date, no coating has proven to have a significant impact on the long-term biocompatibility of MEAs (Bentil and Dupaix, 2018; Mian et al., 2021).

Structural stiffness is directly proportional to the Young’s modulus of the material but also depends strongly on the microelectrode geometry. Long, thin, and flexible MEAs have been fabricated using materials with Young’s moduli ranging from 2 to 5 GPa, such as polyimide (PI), parylene (PPX), and the epoxy-based polymer SU-8 (Tay et al., 2001; Fernández et al., 2009; Hassler et al., 2011; Ordonez et al., 2012; Kim and Meng, 2016; Teo et al., 2016; Weltman et al., 2016; Luan et al., 2017; Musk and Neuralink, 2019; Araki et al., 2020; Mariello et al., 2022). These thin probe geometries provide lower stiffness to reduce the FBR, but they also complicate insertion since they are too flexible to be inserted into the cortex without assistance from needles and shuttles (Weltman et al., 2016; Hanson et al., 2019; Apollo et al., 2020; Atkinson et al., 2022). Furthermore, the surface stiffness of most polymer probes is still limited by their Young’s modulus, which can be 6 orders of magnitude higher than brain tissue, as shown in Figure 1. Lastly, the long-term durability of these polymer probes during *in vivo* implantation has yet to be evaluated extensively, but initial studies suggest they may not meet clinical requirements due to high gas permeability and water uptake (Araki et al., 2020; Mian et al., 2021; Zhang E. N. et al., 2021; Mariello et al., 2022). Consequently, it is advantageous to investigate alternative materials and fabrication techniques for MEA-style implants that could mitigate the adverse effects of mechanical mismatch while ensuring easy insertion.

Carbon nanotube templated microfabrication (CNT-M) is a process that produces patterned CNT composite materials that could meet intracortical MEA clinical requirements (Hutchison et al., 2010; Fazio et al., 2012; Hanna et al., 2014). CNT-M composite structures are formed by growing a patterned carbon nanotube forest and then partially or fully infiltrating the forest with a matrix material to stabilize and strengthen the structure. Chen et al. (2018) fabricated a millimeter tall CNT/carbon composite microelectrode array (we will call this a CNT MEA) with 20 μm diameter probes and a reduced pitch of 100 μm, compared to the UEA’s traditional 400 μm pitch (Figure 1A; Chen et al., 2018). They also demonstrated that the CNT microelectrodes (MEs) are capable of chemical sensing, including the detection of dopamine using fast-scan cyclic voltammetry. Other studies have also shown that CNT composite electrodes are effective for neurochemical detection (Palomäki et al., 2018; de Menezes et al., 2019). While the geometric and chemical detection performance of CNT MEs is promising for intracortical applications, the mechanical properties of the electrodes and their potential impact on biocompatibility have not been evaluated. There is not a common consensus on the biocompatibility of CNTs and CNT composites and evaluating the biocompatibility of CNT composites is beyond the scope of this work. Nevertheless, to be a viable neural implant, the biocompatibility of CNT MEAs should be investigated and future biocompatibility is discussed at the end of this paper.

The aim of this work is to address the following question as the next step in the development of intracortical CNT MEAs: *What range of effective CNT ME moduli can be fabricated, and which moduli within that range will significantly reduce strain on brain tissue during micromotion?* To answer this question, we fabricated CNT MEAs with varying carbon infiltrations to produce microelectrodes with different effective moduli and developed a micromechanical measurement method—the dual deflection (DD) test. The DD is compatible with MEA form factors and can measure a wide range of moduli with a 30% uncertainty. There are many interconnected morphological factors that can influence the initial effective ME modulus and its response to carbon infiltration, including CNT density, spatial distribution of CNT density, carbon deposition rate, and spatial density of carbon deposition. These factors are difficult to quantify and decouple, but the ultimate effect can be simplified down a single hypothesis; that as more carbon is deposited on the existing nanotube structures through carbon infiltration, the nanotubes will thicken and interlock, thereby increasing the modulus of the ME. To quantify the infiltration level, we measured the CNT diameters of the MEs before conducting the DD test. After measuring the range of modulus values fabricated with different infiltration times, we developed a finite element analysis (FEA) model to compare the brain tissue strain induced by a static deflection of probes. The model considered probes with the minimum, median, and maximum effective CNT moduli measured, as well as the modulus of silicon for comparison. The results of the model suggest that CNT MEs with effective moduli in the tens of MPa range, which can be achieved with 15 s of carbon infiltration, could significantly reduce strain on brain tissue. Furthermore, MEs with 15 s of infiltration incurred no apparent damage after deflection in contrast to other infiltration time groups, indicating they may be a more mechanically suitable material candidate for neural probe arrays.

## 2 Materials and methods

### 2.1 CNT MEA fabrication and mechanical testing methods

The fabrication of CNT MEAs was conducted using carbon nanotube microfabrication (CNT-M) methods, which were adapted from Chen et al. (2018). In order to facilitate the mechanical testing in our study, we opted for CNT MEAs with reduced aspect ratios compared to the highest aspect ratio CNT MEs available (as shown in Figure 1A). All MEs were cylindrical with 40 μm diameter and shorter than the 1 mm produced by Chen et al. (2018). Across arrays, the average ME length ranged from 294 ± 11.5 μm to 710 ± 19.6 μm. The effective length of the MEs (*L*_*ME*_), i.e., the distance from the substate to the point of wire contact, ranged from 200 ± 5 μm to 562 ± 25.2 μm and were the values used for the modulus calculations. Similarly, the effective length of the wire (*L*_*w*_) was also recorded for each measurement and used for the modulus calculations. Additionally, we increased the pitch between MEs within our arrays from 100 to 400 μm (same as the UEA) to provide a larger area for mechanical testing. Due to the lower aspect ratio, the MEAs could be grown with a reasonable yield as vertical freestanding structures up to approximately 600 μm without requiring a support grid or plasma etching. The lengths of the MEs were measured using optical microscopy, with an uncertainty of ± 5 microns. MEs exhibiting non-vertical CNT growth, leading to misalignment between their tips and base when viewed perpendicular to the substrate, were excluded from the study. Following the growth step, two subsets of arrays underwent separate carbon infiltration runs for 15 and 30 s to increase their effective modulus. Carbon infiltration was accomplished using a “1” quartz tube CVD furnace at 900°C, with ethylene and hydrogen flow rates of 337 and 310 sccm, respectively, and argon flow during ramp up and cooldown. To aid in the characterization of the infiltration and growths, the CNT diameters were measured by scanning electron microscopy (SEM) at a distance of one micron from the base of the MEs prior to mechanical testing. An average of 27 nanotube measurements were taken per ME.

**FIGURE 2:**
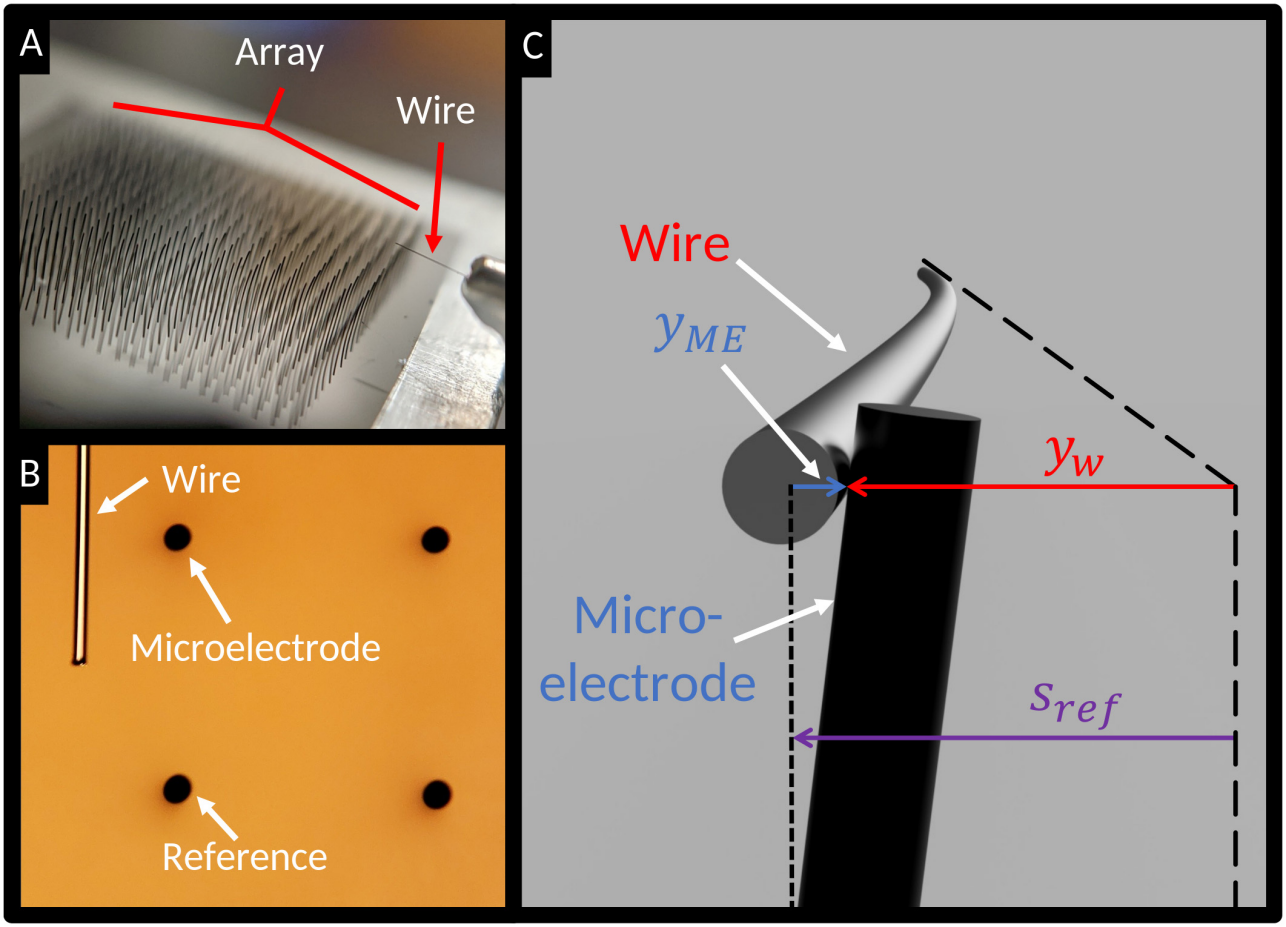
Overview of the dual deflection test to measure the effective modulus of CNT microelectrodes. **(A)** The CNT array and the testing wire used to deflect the CNT microelectrodes. **(B)** The top-down microscope view of the wire and probes used to measure the deflections. The reference electrode is used to measure the total distance the substrate moves, s_ref_. **(C)** A 3D representation of the wire and CNT microelectrode interaction. Distances y_ME_ and y_w_ are the microelectrode and wire deflections, respectively. s_ref_ is the total displacement of the array relative to the wire, as measured by the reference electrode in **(B)**.

In contrast for the high-aspect-ratio microelectrodes studied here, transverse loading is expected to produce primarily bending deformation rather than shear deformation. However, the dimensions of the CNT microelectrodes are too small for traditional 3-point bending. As a result, mechanical testing was conducted using a method referred to as Dual Deflection (DD). The DD test uses deflections of two simple cantilever beams in contact with one another—one cantilever with a known stiffness (a microwire, as shown in Figure 2A and one with an unknown stiffness (the CNT ME)—to determine the effective Young’s modulus of the unknown beam. The known beams used for testing were straight segments of Stableohm 650 wire (California Fine Wire), ranging in length from 2.55 to 4.6 mm ± 10 μm. The wire was secured with adhesive to a larger wire for handling and mounting to a 3-axis stage (Figure 2A). Deflections were performed under an optical microscope and recorded for later analysis (Figures 2A,B). The wire is fixed in space relative to microscope objective and the array rests on the microscope stage, which is then moved laterally to force the ME into the wire. Since both the wire and ME deflect, the position an independent reference ME must be recorded to decouple the deflection distances. The difference between the deflection of the wire, denoted as *y_w_*, and the reference displacement, denoted as *s*_*ref*_, represents the deflection of the microelectrode (*y*_*ME*_). A 3D representation illustrating this interaction is shown in Figure 2C. The deflections (*y*_*w*_, *y*_*ME*_), the modulus of the wire (*E*_*w*_), the diameter of the ME and wire (*d*_*ME*_, *d*_*w*_), and the length of the ME and wire (*L*_*ME*_, *L*_*w*_) are used in the Euler-Bernoulli beam theory equation with a circular cross section (Equation 1) to calculate the modulus of the ME (Equation 2) as follows:


$$E=\left(\frac{F}{y}\right)\,\frac{64\,L^{3}}{3\,\pi \,d^{4}}$$ (1)

$$E_{M\,E}=\left(\frac{E_{w}}{y_{M\,E}}\right)\,\left(\frac{d_{w}^{4}\,L_{M\,E}^{3}}{d_{M\,E}^{4}\,L_{w}^{3}}\right)\,y_{w}$$ (2)

In this work, the CNT ME is approximated as a homogeneous beam such that the DD test measures the effective modulus of the composite material under small deflections, which aggregates the anisotropic morphological effects of the CNT forest growth and carbon infiltration into a single value that is proportional to the effective stiffness of an implanted probe under similar bending conditions.

The DD test concludes when the ME fails or deflects to a point where the wire slips off. To ensure that the ME deflections reached a minimum of 10 μm before the wire slipped off, the wire contacted the ME at an average distance of 175 ± 75 μm from the top of the ME. The DD test was determined to measure effective modulus with 30% accuracy. Verification was achieved by performing the test with two microwires with known geometry and modulus.

### 2.2 Finite element analysis methods

To model the potential impact of CNT ME micromotion on brain tissue strain at the probe-tissue interface, a first order finite element analysis (FEA) model was used as a comparative benchmark between CNT MEs and a silicon probe of identical geometry. The real mechanical interactions between a chronically implanted brain probe and brain tissue are complex, dynamic, and not fully understood (Lecomte et al., 2018). A truly representative model is beyond the scope of this work and would need to include accurate representations of the different mechanical properties of the glial sheath and healthy neural tissue, the anisotropic properties of the glial sheath, the viscoelastic properties of the brain, interfacial tissue-biomaterial adhesion, the anisotropic properties of the CNT probes, and various cyclical micromotion modes. We applied the following simplifications and assumptions, which are in line with previously published neural probe micromotion models (Lee et al., 2005; Subbaroyan et al., 2005; Polanco et al., 2014, 2016): Micromotion was simulated with a static lateral probe deflection of 10 μm at the brain surface, which is on the scale of breathing induced micromotion in rats (Sridharan et al., 2015). For these models we used the highest aspect-ratio CNT MEs geometries fabricated by Chen et al. (2018)—1,000 μm long and 20 μm in diameter—as these are more appropriate geometries for intracortical probes. We included a 10 μm radius fileted tip (not used by Chen et al., 2018) to avoid stress concentrations in the model due to sharp edges. The brain was modeled as a 1,500(x) × 1,500(y) × 400(z) μm cuboid. The probe-brain interaction was modeled as a half-symmetry model, with half of the probe diameter embedded in the brain from the surface to a depth of 1,000 μm. Following the assumptions made by Sabboroyan *et al.*, we modeled the brain tissue as an isotropic elastic material instead of viscoelastic because the deflections and strain rates are small enough to treat the brain tissue as linearly elastic, in a first order approximation. The brain tissue was assigned a Young’s modulus of 6,000 Pa and was assumed to be incompressible with a Poisson’s ratio of 0.48, which aligns with the previously cited FEA modeling studies on probe-brain interactions. The probe was modeled with a Poisson’s ratio of 0.2. To explore the full range of effective moduli achievable with CNT MEs, we considered the maximum, median, and minimum measured CNT ME effective moduli obtained in this study, in addition to the modulus of silicon for comparison. The surfaces of both the probe and the brain tissue were connected, preventing slippage or separation, with the probe designated as the primary surface and the brain tissue as the secondary surface. Fixed boundary conditions were applied to the lateral sides of the brain cuboid, while the front and back faces were also assigned fixed boundary conditions to maintain the integrity of the half symmetry model.

## 3 Results

### 3.1 Dual deflection results

**FIGURE 3:**
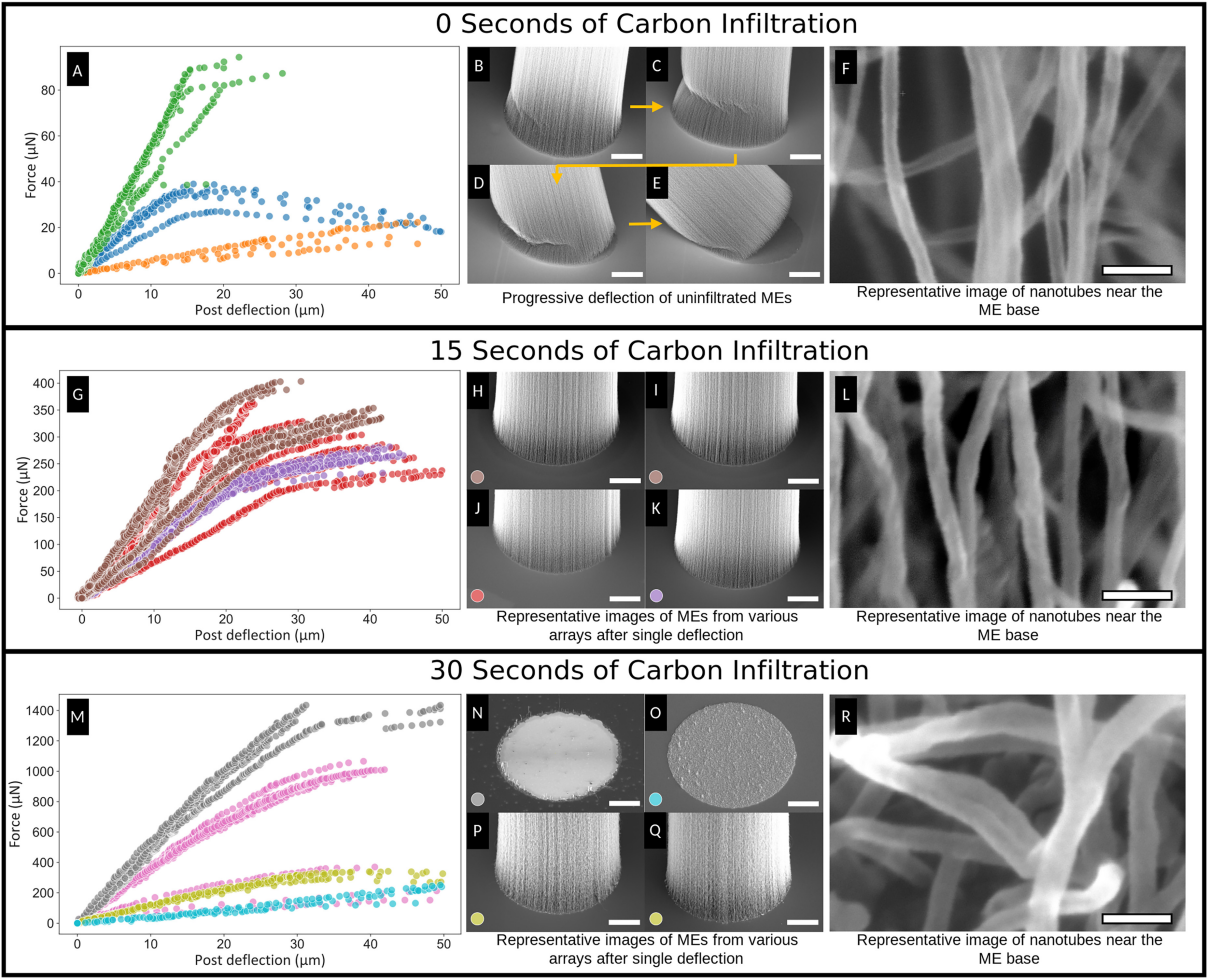
Summary of all dual deflection data. Data is separated by infiltration level. For each level, we show the following data: a force deflection graph of all the measured microelectrodes, where data from each array is shown in a different color **(A,G,M)**; SEM images of the microelectrode bases after a single deflection (**B–E,H–K,N–Q** Scale bar = 10 μm); a close-up image of the nanotubes in the MEs **(F,L,R.** Scale bar = 50 nm). Microelectrodes shown in **(B–E)** were progressively deflected to **(B)** 15 μm, **(C)** 45 μm, **(D)** 160 μm, and **(E)** 210 μm. The colored dots on images **(H–K,N–Q)** indicate the array of origin, matching the corresponding array color in the force deflection curves.

Across 10 arrays, a total of 64 microelectrodes were tested with the DD method until failure or slippage occurred, and were examined under SEM (Figure 3). The duration of carbon infiltration exhibited discernible effects on the mechanical behavior, failure modes, and nanostructure of the CNT MEs. The force deflection curves of all these microelectrodes are shown in Figures 3A,G,M up to 50 microns of deflection. The mechanical behavior was generally non-linear over large deflections of 50 microns, but larger interaction forces were achieved with increased infiltration time. Furthermore, increased carbon infiltration corresponded to an increase in the nanotube diameter, as evidenced in Figures 3F,L,R. Infiltration time also had an impact on the failure mode behavior at the base of the MEs (Figures 3B–E,H–K,N–Q). Scanning electron micrographs were taken of a subset of the deflected MEs for each infiltration time. MEs without infiltration exhibited buckling and compression-induced collapse even at deflections as small as 15 microns (Figure 3B). Larger deflections, up to 210 microns, tore the nanotubes under tension and left a short “carpet” of nanotubes on the substrate (Figures 3D,E). In contrast, some MEs with 30 s of infiltration cleanly separated from the surface, leaving little residue behind (Figure 3N). However, clean separation was not consistently observed, as some MEs with 30 s of infiltration still exhibited the presence of a “carpet” (Figure 3O), while others appeared undamaged (Figures 3P,Q). Of all the infiltration times, microelectrodes with 15 s of infiltration displayed the best post-deflection integrity, with no observable damage seen in any of the imaged microelectrodes from this group (Figures 3H–K).

**FIGURE 4:**
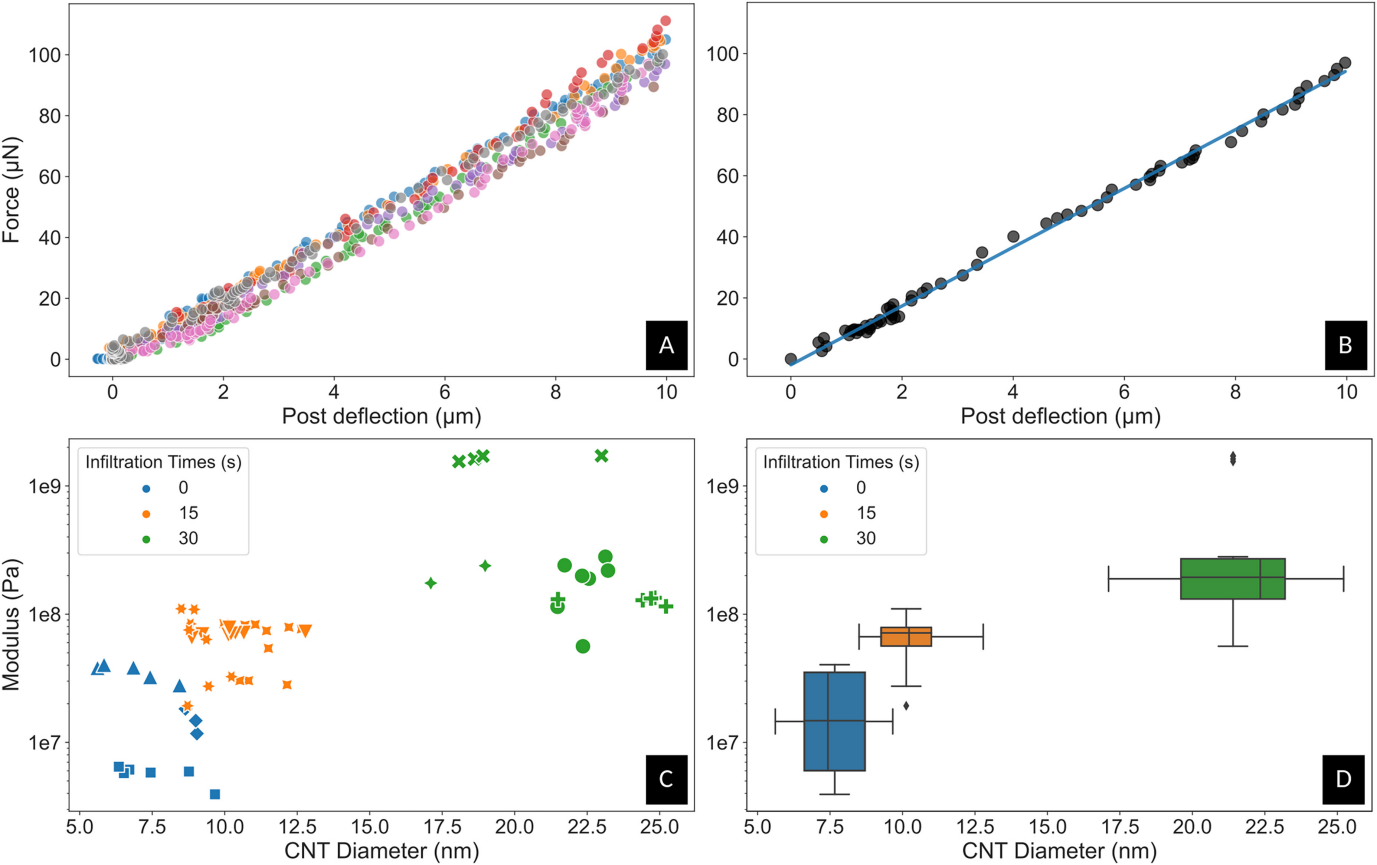
Processed data from the dual deflection test. **(A)** Force deflection data of multiple microelectrodes on a single array truncated to 10 microns of deflection. Colors differentiate microelectrodes. **(B)** The total least squares (TLS) fit of a single ME deflection. **(C)** Effective modulus vs. CNT diameter. The effective modulus is calculated with the slope of the TSL fit in **(B)** and Equation 2. Log scale of effective Modulus vs. linear scale of CNT diameter. Each data point represents a single post, and the shapes indicate arrays. The colors indicate infiltration time. **(D)** Dual boxplots of the effective Modulus vs. CNT diameter grouped by infiltration time. Outliers are defined as larger than 1.5 times the interquartile range.

To avoid non-linear behavior for the quantitative analysis, the force deflection curves were truncated to the physiologically relevant range of brain micromotion, about 10 microns (Lee et al., 2005; Gilletti and Muthuswamy, 2006). Example truncated deflections from a single array are shown in Figure 4A. To improve the quality of fits in the first 10 microns of deflection, we established a criterion of 8 minimum data points which filtered out 5 microelectrodes from the full dataset—one from Figure 3A and four from Figure 3M—reducing the dataset used to calculate the moduli seen in Figure 4C to 59 microelectrodes. The goodness of the total least squares fits was quantified with the reduced Chi squared and R squared values, which were calculated assuming an uncertainty of three microns in wire and microelectrode deflections. The Chi squared values ranged from 0.0012 to 0.017 and the R squared values ranged from 0.95 to 0.99, indicating good linear fits and a probable overestimation of the deflection uncertainty. An example fit is shown in Figure 4B.

Images like Figures 3F,L,R were used to measure the average CNT diameter for each microelectrode, which is plotted against modulus in Figure 4C. In that figure, the data are grouped by shape according to array and by color according to infiltration time. Contrary to our hypothesis, effective modulus data within each array and within each infiltration time group did not show a clear trend with increasing CNT diameter. However, dual-axis boxplots for CNT diameter and effective modulus per infiltration time (Figure 4D) show that increased infiltration time correlates positively with modulus and CNT Diameter, thus supporting our hypothesis. We verified the uniqueness of each infiltration time group with a 3-way Kruskal-Wallis (ANOVA) test, which produced a *p*-value of 1.5e-10 when comparing effective modulus and a *p*-value of 5.4e-12 when comparing CNT diameter. Out of the three infiltration times, the 15 and 30 s groups had modulus outliers, shown on Figure 4D as diamond markers. Outliers were defined as > 1.5 times the interquartile range. The 15 s infiltration time group had one low modulus outlier and the 30 s infiltration time group had 4 high modulus outliers, the latter of which all came from the same array. None of the average CNT diameters were outliers. The outlier MEs were excluded from the mean, standard deviation, sample size, standard error, and coefficient of variance calculations. Thus, the total ME sample size used for averages and statistical quantifications is 54. All values described in the following paragraph use this data subset, i.e., all measured MEs except the outliers.

To quantify the variance in effective modulus and carbon nanotube diameter and discern any trends based on infiltration time or array, we employed two grouping methods: (1) grouping MEs by infiltration time only, and (2) grouping MEs by array only. The results of each grouping are described below, but only the results from group 1 are reported in Table 1. With group 1, the average CNT diameter and average effective Young’s modulus increased with infiltration time, as hypothesized. The means and standard deviations of the CNT diameters and moduli per infiltration group are shown in Table 1. The high degree of mechanical inconsistency between arrays, while undesirable, is not totally unexpected, given the limitations of the lab-scale furnaces used for growth in this initial mechanical characterization study. Insight into these limitations and proven solutions can be found in the discussion section. The CNT diameter results in group 1 were less varied than the effective modulus results as evidenced by the coefficient of variance (CV). The CV is a measure of how wide a distribution is relative to its mean and is useful to compare distributions with different units and scales. It is calculated as the ratio (or percent) of the standard deviation (σ) over the mean (μ).

**TABLE 1:** Statistical quantification of microelectrodes’ effective moduli and CNT diameters, excluding outliers > 1.5 times the interquartile range.

Infiltration time	0 s	15 s	30 s
Number of MEs measured	15	25	14
Modulus: mean	19.6 MPa	67.7 MPa	168 MPa
Modulus: standard dev (SD)	14.5 MPa	22.7 MPa	62.3 MPa
Modulus: CV	74%	34%	37%
Modulus: standard error (SEM)	3.76 MPa	4.55 MPa	16.7 MPa
Number of CNTs measured	322	784	338
CNT diameter: mean	7.5 nm	10.2 nm	22.6 nm
CNT diameter: standard dev (SD)	1.6 nm	2.0 nm	3.3 nm
CNT diameter: CV	22%	20%	15%
CNT diameter: standard error (SEM)	0.09 nm	0.07 nm	0.20 nm

MEs were grouped by infiltration time for quantification.

Where σ is the standard deviation and μ is the mean. Across the three infiltration groups, the effective modulus CVs ranged from 34 to 74%, while the CNT diameter CVs only ranged from 15 to 22%. This trend held upon examining the variability of effective ME moduli and CNT diameters within each array, i.e., group 2. The effective modulus CVs per array ranged from 4.8 to 55%, while the CNT diameters CVs per array only ranged from 12 to 22%. Separating the array data by infiltration time gives insight into the variability between arrays fabricated with each infiltration time. The CNT diameters CVs of the uninfiltrated arrays had the widest range, 15–22%, but the effective modulus CVs were the least varied, ranging from 13 to 22%. The converse was true for arrays fabricated with both 15 and 30 s of infiltration. The effective modulus CVs ranged from 5 to 46% and 6–41% for the 15 and 30 s infiltrations, respectively. However, the CNT diameter CVs only ranged from 17 to 18% and 12–15% for the 15 and 30 s infiltrations, respectively. Finally, a few observations of note concerning the outliers. Outliers were excluded only from the summary statistics in Table 1; they were retained for the modulus *range* reported throughout the paper and were used directly as the “CNT Maximum” (1.7 GPa) case in the FEA. Out of the ME outliers that were excluded, 4 out of 5 originated from the same array, which is indicated in Figure 4D with green “X”s. These outliers represented the highest moduli recorded in this study, with an average of 1.65 GPa. Notably, this array also had the most tightly grouped effective modulus results, with the lowest CV at 4.8%, and a CNT diameter CV of only 15%. Nevertheless, we excluded them from our statistical quantification of the infiltration groups because they were 10x stiffer than the average 30 s infiltrated array, indicating some abnormally large variation during fabrication.

### 3.2 Finite element analysis results

**FIGURE 5:**
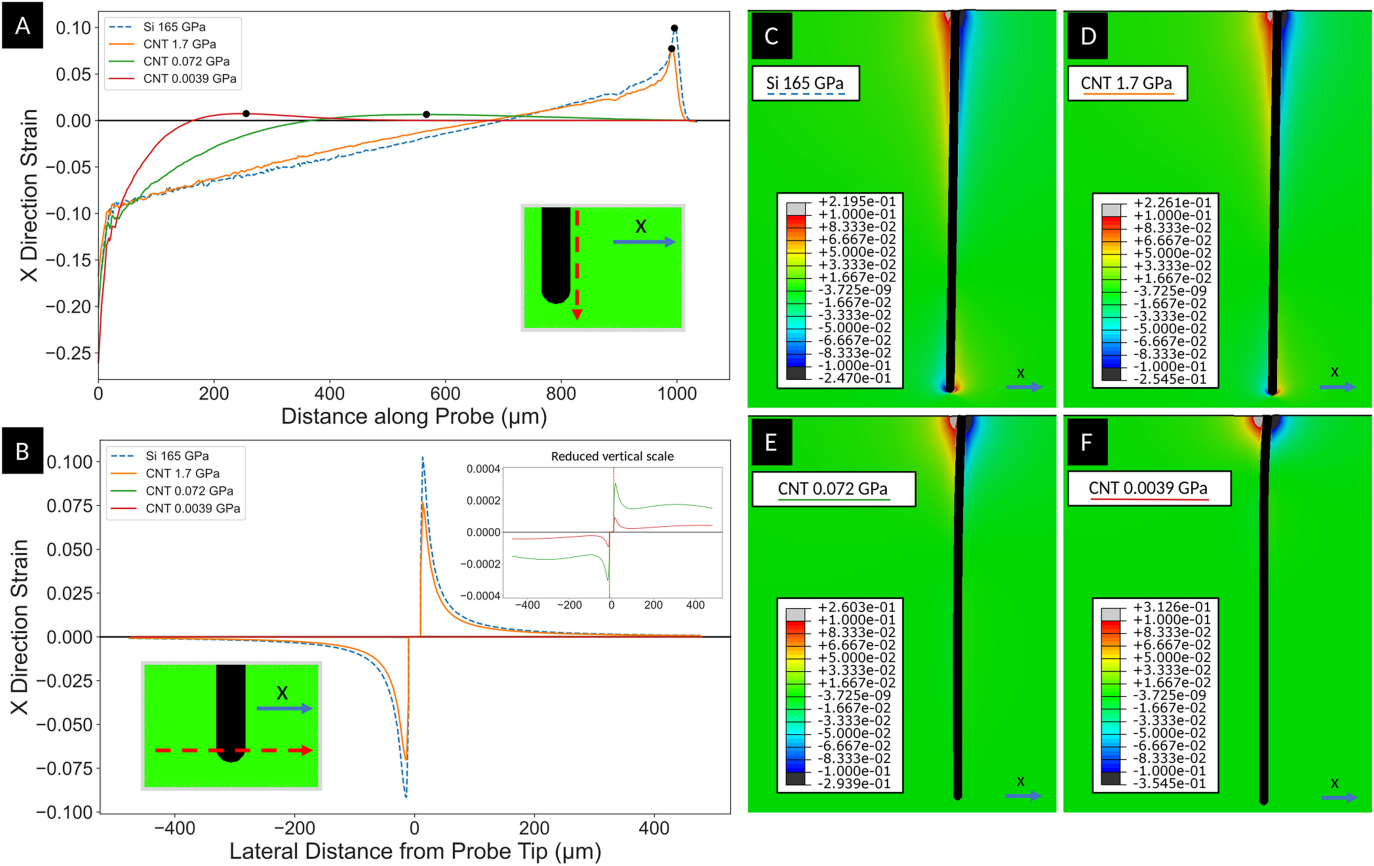
FEA model of brain tissue strain when the top of the ME is deflected 10 μm. Strain and deflection are both in the positive X direction, as indicated by the blue arrows. The maximum, minimum, and median effective moduli of CNT MEs recorded in this study and the modulus of silicon are used. **(A,B)** Measurement profiles of the strain heatmaps shown in **(C–F)**. **(A)** The strain profile measured parallel to the probe on the right side as indicated by the red dashed arrow. The black markers indicate the point of maximum compression. **(B)** The strain profile measured perpendicular to the ME and 10 μm above the end of the probe, as indicated by the red dashed arrow. The segment of zero strain near 0 is where the measurement line passes through the ME. To visualize the small strains of the lower two moduli, a zoomed in graph is shown in the upper right-hand corner. The heat maps of brain strain with deflected, implanted probes are show with **(C)** a 165 GPa silicon probe, **(D)** a 1.7 GPa CNT probe, **(E)** a 0.072 GPa (72 MPa) CNT probe, and **(F)** a 0.0039 GPa (3.9 MPa) CNT probe.

To determine whether probe tip strain could be reduced using CNT MEs, FEA modeling was performed using the full range of CNT ME effective moduli that we measured (Figure 4C), including the outliers. We simulated probe deflection and brain tissue strain for probes with moduli matching the maximum (1.7 GPa), median (72 MPa), and minimum (3.9 MPa) effective CNT ME moduli, as well as one matching modulus of silicon (165 GPa) for comparison. The CNT ME effective moduli and their comparison to the modulus of silicon is shown in Table 2. The FEA model results are show in Figure 5, which has three main parts: a graph of tissue strain along the length of the probe at the probe-tissue interface (a), a graph of tissue strain measured laterally from the probe tip (b), and the strain heatmaps of the deflections (C–F).

**TABLE 2:** FEA results summary on induced brain tissue strain.

Probe	Modulus (E)	$\frac{E}{E_{S\,i}}$	Characteristic decay length	$\frac{\epsilon_{t\,i\,p}}{\epsilon_{t\,i\,p,S\,i}}$
Silicon	165 GPa	1	–	1
CNT maximum	1.7 GPa	1e-2	–	0.77
CNT median	72 MPa	4e-4	312 μm	3.3e-3
CNT minimum	3.9 MPa	2e-5	139 μm	9.9e-4

Characteristic strain decay lengths—measured from the probe displacement at the brain surface to the zero-strain point—are shown. Because the Si and CNT Maximum probes were too stiff at this length to exhibit bending, the decay length could not be measured. The last column shows the ratio of brain tissue strain at the tip compared to a silicon probe, ϵ_tip_/ϵ_tip,*Si*_.

When the probes were deflected laterally at the surface, the two higher-modulus probes largely exhibit rotation (heatmaps C–D) while the two lower-modulus probes primarily exhibit bending (heatmaps E–F). The rotation of the 165 GPa and 1.7 GPa probes is evidenced in their heatmaps by the switch of strain direction at the tip compared to the surface. At the surface of the brain, the probe is deflected to the right, which puts the tissue under compression on the right side of the probe, indicated by the blue region on the heatmap. The magnitude of the tissue strain decreases until a point of zero-strain is reached. Beyond the zero-strain point, the strain is inverted, inducing a rise in tensile strain on the right side of the probe. At the tip of the probe, there is substantial strain. Unlike the stiffer probes, the 72 MPa and 3.9 MPa probes primarily exhibit bending as opposed to rotation. The bending of the probes can be seen in their heatmaps near the tissue surface (Figures 5E,F) and is further evidenced by the lack of visible strain beyond the bending region.

To quantify the differences in tissue strain induced by the rotation and bending behavior of the two stiff and the two flexible probes, respectively, strain was measured from the tissue surface to the probe tip along a vertical path on the right side of each probe. The results of these measurements are shown in Figure 5A. The features visible in the heatmaps of the stiff probes are clearly represented in the graph (going from tissue surface to probe tip); an initial decrease in strain to a point of zero-strain, after which the strain switches signs and increases to substantial strain at the tip. Additionally, there are several finer features that are revealed in this graph: (1) a rapid drop in the strain at the tissue surface due to stress concentration, (2) a nearly linear change in strain going through the zero-strain point and extending almost to the tip, and (3) a spike in strain at the tip due to stress concentration. At the spike, the strain induced by the 1.7 GPa probe reaches ∼77% of the tip strain induced by the 165 GPa probe. On the other hand, the strain profiles induced by the flexible 72 MPa and 3.9 MPa probes, away from the tissue surface, are small enough to not be visible on the heatmaps but are evident in the graphs. The induced strain is large at the surface but experiences a rapid spatial decay to zero, followed by a small increase in positive strain (up to ∼3% of the initial surface strain) before decaying toward the tip. The local maximum after the zero point is indicated by a black dot in Figure 5A. To quantify how rapidly the strain decreases in the two flexible probes, we define a spatial decay length as the distance from the tissue surface to the zero-strain point and report these spatial decay lengths in Table 2.

To quantify strain in the region of the probe tip—where neural recording most commonly occurs—Figure 5B examines the tissue-strain profiles 460 μm lateral to the probe tip in each direction. The zero-strain region in the middle of the graph is where the measurement line passes through the probe tip. The 3.9 MPa CNT probe is ∼50,000x less stiff than the Si probe and induces a peak tip strain ∼1000x times smaller. To visualize the wide range of strains of the 72 and 3.9 MPa probes, Figure 5B has an inset graph with a strain scale 250 times smaller. The ratios of induced tip strain compared to the silicon probe are shown in Table 2.

## 4 Discussion and conclusion

The requirements for an effective biomaterial for neural implants are vast and varied. In this study, we narrowed our focus to one biomaterial and one requirement: the mechanical compatibility of high aspect ratio CNT microelectrodes. Thus, the questions motivating this study were:

1. *What range of effective CNT ME moduli can be fabricated?*
2. *Will moduli within that range significantly reduce strain on brain tissue during deflection?*

Using the novel dual deflection method, we were able to measure the average effective modulus of the uninfiltrated CNT MEs as 19.6 MPa and the average effective modulus of the 900°C, 30-s infiltrated CNT MEs as 168 MPa, a range of ∼8.5x. If we examine individual MEs, including outliers, we demonstrated that CNT MEs can have effective moduli as low as 3.9 MPa and as high as 1.65 GPa, a range of almost 500x. For reference, this modulus spans the range from modulus of polyimide (∼2.5 GPa), a popular choice for flexible polymer neural probes, down to crosslinked PDMS (∼3.7 MPa), which has been used in contact lenses (Stieglitz et al., 2000; Rousche et al., 2001; Takeuchi et al., 2003; Cheung et al., 2007; Wang et al., 2014; Musk and Neuralink, 2019).

To answer the second question, we used finite element modeling of probes with the bending moduli of silicon and CNT MEs. The silicon and CNT probes with moduli in the GPa range induced strain along the entire probe, including the tip, while the two lower modulus probes—3.9 and 72 MPa—showed no significant strain at the probe tip. Micromotion induced strain has been shown to activate microglia and astrocyte inflammatory responses (Karumbaiah et al., 2012), and probes in the tens-of-MPa range have been shown to significantly reduce the neuroinflammatory response during chronic implantation in rats (Nguyen et al., 2014; Lecomte et al., 2018; Stiller et al., 2018). Thus, CNT probes with low carbon infiltration levels could potentially reduce the mechanically induced FBR caused by micromotion of the neural probe tip. However, we acknowledge that even if the *in vivo* reduction in brain tissue strain is comparable to our model, the chronic FBR may still be significant due to mechanical and non-mechanical triggers not represented in our model. Lastly, achieving significant strain reduction with carbon infiltrated microelectrodes is potentially beneficial as even the minimal 15 s infiltration makes the probes less susceptible to damage during handling and may help the probes survive insertion without assistance.

### 4.1 Capabilities and uncertainties of dual deflection method

The dual deflection method proved to be effective at rapidly measuring vertical microelectrodes across the wide range of effective moduli produced in this study. Moreover, an even wider range of measurements is possible by further adjusting the length of the testing wire. The DD test also proved effective for vertical structures that were traditionally difficult to measure, i.e., too large for AFM and too small for traditional bending tests. With the DD test, tens of vertically aligned microelectrodes can be tested in minutes without needing to modify the array.

The 30% absolute effective modulus uncertainty of the DD measurements was deemed adequate for the effective moduli measurements in the present work. DD measurement uncertainty can be further reduced if needed. DD test uncertainty, which is dominated by wire cantilever compliance uncertainty, could be reduced with cantilever calibration techniques, such as the Sader method (Sader et al., 1999). Since all DD measurements on our arrays were performed with testing wires from the same spool, the resulting absolute calibration error would be a systematic error impacting each measurement similarly.

Additionally, there may be random measurement-to-measurement errors in the DD method caused by focusing differences, computer vision tracking errors, and/or deflection rates. If the DD method random error was the dominant source of variation, we would expect similar electrode-to-electrode variation within each array. However, we see significantly different variations within each array, with some arrays having mechanically consistent MEs and others having a wide spread of effective ME moduli, such as the 15 s infiltration group in Figure 4C. This indicates that the effective modulus variations seen across arrays and infiltration times (the datapoints in Figure 4C) reflect real mechanical differences instead of random measurements errors.

### 4.2 Discussion of effective CNT ME modulus results

The material and mechanical behaviors of the carbon infiltrated CNT forests merit further scrutiny. Our hypothesis, based on work at longer infiltration times, was that even the short carbon infiltration times used here would begin to lock together and thicken the nanotubes, both of which would increase the effective ME modulus. Comparison of the different time groups showed that the effective modulus distributions were distinct from one another, as evidenced by the lack of overlap between the interquartile ranges and an ANOVA test (Figure 4D). The CNT diameter distributions of each infiltration time were also unique. Overall, the data supported the hypothesis of effective modulus and CNT diameter increasing with infiltration time. When MEs were grouped and averaged by infiltration time, the average effective modulus and average CNT diameter both increased with infiltration time.

In contrast, within each time group, we did not see a correlation between effective modulus and measured CNT diameter. We identify two potential reasons why the relationship between CNT diameter and effective modulus was not observed within a single time group or within a single array:

1. The method for measuring CNT diameter was insufficient to characterize the full distribution of CNT diameters in each electrode, and
2. CNT forest morphology, which is difficult to characterize, can contribute to the effective modulus independently of, or combine with CNT diameter for second order effects, which may dominate.

Regarding point 1, electron microscopy of the outer electrode surface was used for CNT diameter measurements. While SEM enables high-resolution imaging of individual nanotubes, it limits measurements to small sampling areas on the outer edge of the forest, with no effective way to measure the potential spatial gradient of carbon deposition and forest density. Diameter measurements were performed on approximately 20–30 nanotubes for each microelectrode (ME), resulting in a total of 1,444 diameter measurements across all arrays. These local samplings represent a very small fraction of the nanotubes in each ME, which could result in diameter averages that are not representative of the CNT diameters in the ME. On point 2, CNT forest morphology—including factors such as CNT crystallinity, alignment, and spacing—is sensitive to growth conditions, including temperature, iron thickness, catalyst pattern, gas flow rates, and local gas composition, (Plata et al., 2009; Yasuda et al., 2009, 2011; Chen et al., 2015) which can lead to significant CNT forest sample-to-sample variation even under quite similar growth conditions. Variations in morphology can exist even across a single sample (Meshot and Hart, 2008; Bedewy et al., 2011; Oliver et al., 2013). We suspect that most relevant as-grown morphologies for this work are the CNT forest density and its spatial distribution. Beyond impacting the initial effective modulus, these factors can impact gas diffusion, which is a significant mechanism during carbon infiltration at 900°C. The diffusion rate impacts the carbon deposition rate and the spatial carbon infiltration distribution and thus influences the degree and spatial distribution of CNT interlocking. These morphological variations could contribute to the variations in mechanical properties we observed. There is significant room for improving the growth and infiltration uniformity at scale compared to that seen in our study. Our supported catalyst CNT growths were performed in a 25 cm × 2.5 cm tube furnace which results in significant temperature and gas composition gradients. However, our lab scale furnaces are much smaller than those used for industrial scale CNT growth. For example, uniform growth over large areas by supported catalyst CVD has been demonstrated using a 6 m × 1.5 m lateral batch reactor and shower head gas delivery configuration (Yasuda et al., 2009). In our reactor, the temperature and gas composition gradients will also have a large impact on carbon infiltration rate which could be improved by using larger reactors.

### 4.3 Future work on CNT MEA biocompatibility

The CNT MEs are a mechanically promising material for further evaluation in intracortical neural implants applications, but the ultimate biocompatibility of the material remains unknown. In existing literature, the biocompatibility of CNTs is not well defined and contradictory results are often reported. The heterogeneity of CNT structures makes it difficult to generalize one CNT toxicity study to CNTs produced under different conditions. Many studies in the 2000s and early 2010s reported cytotoxic and carcinogenic properties to individual CNTs, especially upon pulmonary inhalation (Johnston et al., 2010; Popescu et al., 2013; Razavi and Khandan, 2017; Li and Cao, 2018). But by the late 2010s a more nuanced picture of CNT biotoxicity was revealed as the understanding of factors inducing cytotoxicity matured, and mitigating methods, such as functionalization, were discovered (Kostarelos, 2008; Razavi and Khandan, 2017; de Menezes et al., 2019; Redondo-Gómez et al., 2019; Heller et al., 2020). Additionally, researchers began investigating the biocompatibility of implanted CNT structures, which were often better received by the body than unstructured CNTs that were injected or inhaled (Nayagam et al., 2011; Sharma and Madou, 2012). Furthermore, the cytotoxicity of trace catalyst metals used for patterned CNT structures, such as the iron used in this work, may also be mitigated by carbon encapsulation or have a negligible effect (Palomäki et al., 2018). Some works even reported no toxicity or biocompatibility with mammalian biology (Huczko and Lange, 2001; Dubin et al., 2008; Kishore et al., 2009; Harreither et al., 2013; Vega-Estrada et al., 2016). For example, some studies have reported that CNTs promote neurite growth and neuron cell adhesion, improving neural recording signals (Hanein and Bareket-Keren, 2013). Considering the diversity of CNT properties and toxicity results, the biocompatibility of the CNT MEA, with each of the infiltration levels, must be evaluated independently by *in vitro* cell viability experiments and chronic *in vivo* implantations in small and large animals. In conclusion, using CNT-M and three levels of carbon infiltration, we were able to fabricate carbon nanotube microelectrodes with effective moduli ranging from 3.9 to 1.7 GPa. When MEs were grouped and averaged by infiltration time, the average effective modulus and average CNT diameter both increased with infiltration time. FEA modeling demonstrated that probes with moduli at 72 MPa and below showed no significant strain at the probe tip when micromotion was simulated. This suggests that CNT MEs with minimal infiltration may prove interesting for intracortical probe applications.

## Data Availability

The raw data supporting the conclusions of this article will be made available by the authors, without undue reservation.
